# Recent Developments of 1,3,4-Thiadiazole Compounds as Anticancer Agents

**DOI:** 10.3390/ph18040580

**Published:** 2025-04-16

**Authors:** Serena Indelicato, David Bongiorno, Manuela Mauro, Stella Cascioferro

**Affiliations:** Department of Biological, Chemical, and Pharmaceutical Sciences and Technologies (STEBICEF), University of Palermo, Via Archirafi 32, 90123 Palermo, Italy; serena.indelicato@unipa.it (S.I.); david.bongiorno@unipa.it (D.B.); manuela.mauro01@unipa.it (M.M.)

**Keywords:** 1,3,4-thiadiazoles, anticancer agents, breast cancer, pancreatic ductal adenocarcinoma, sulfur-containing heterocycles

## Abstract

The World Health Organization has recently underlined the increasing global burden of cancer, with a particularly alarming impact on underserved populations. In recent years, 1,3,4-thiadiazole has emerged as a versatile pharmacophore to obtain bioactive compounds. The pharmacological properties of this ring are primarily attributed to its role as a bioisostere of pyrimidine, the core structure of three nucleic bases. This structural feature endows 1,3,4-thiadiazole derivatives with the ability to interfere with DNA replication processes. Additionally, the mesoionic behavior of this heterocycle gives it important properties, such as the ability to cross biological membranes and interact with target proteins. Noteworthy, in analogy to the other sulfur heterocycles, the presence of C-S σ* orbitals, conferring small regions of low electron density on the sulfur atom, makes interaction with the target easier. This review focuses on the most promising anticancer agents with 1,3,4-thiadiazole structure reported in the past five years, providing information that may be useful to medicinal chemists who intend to develop new anticancer derivatives.

## 1. Introduction

**Figure 1:**
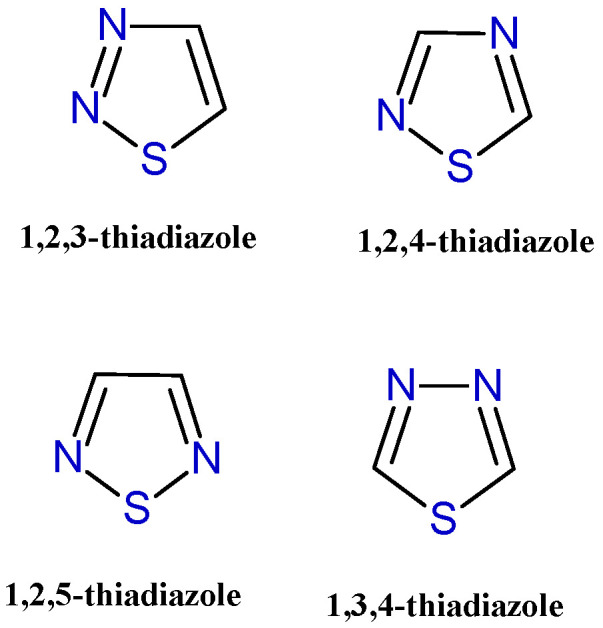
Chemical structures of the four thiadiazole isomers.

Heterocyclic rings are commonly found in bioactive molecules and are considered important pharmacophores in the development of new drugs with various therapeutic properties [1]. Their significance in drug design is attributed to their ability to influence the physicochemical properties, biological activity, pharmacokinetics, and safety profiles of the compounds they form part of [2]. Among the numerous heterocycle derivatives described for their potent therapeutic properties, here we focus on the five-membered sulfur-containing heterocycle thiadiazole. The presence of a sulfur atom gives it promising characteristics for the development of bioactive molecules. In particular, the low-lying C-S σ* orbitals in these compounds generate localized regions of low electron density on the sulfur atom (known as σ-holes), which may contribute to interactions with biological targets [3,4]. Thiadiazole may exist in four isomeric forms: 1,3,4-thiadiazole, 1,2,3-thiadiazole, 1,2,4-thiadiazole, and 1,2,5-thiadiazole (Figure 1), with 1,3,4-thiadiazole being the most extensively studied isomer, demonstrating the most promising therapeutic activities.

**Figure 2:**
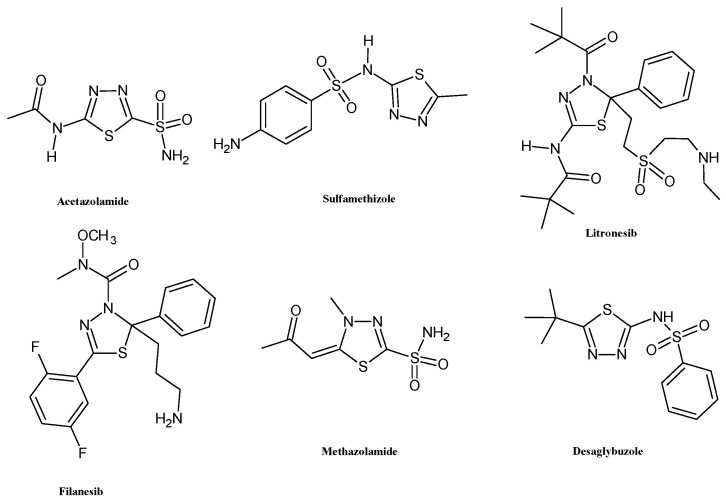
Chemical structures of FDA-approved drugs bearing 1,3,4-thiadiazole scaffold.

The biological properties of this ring are primarily attributed to its role as a bioisostere of pyrimidine, the core structure of three nucleic bases. This structural feature endows 1,3,4-thiadiazole derivatives with the ability to interfere with DNA replication processes [5]. Additionally, the mesoionic nature of the 1,3,4-thiadiazole ring enhances the capacity of these heterocyclic compounds to cross cellular membranes and bind to biological targets, contributing to their good oral absorption and bioavailability [6]. These properties have encouraged the use of the thiadiazole scaffold for the development of several FDA-approved drugs including acetazolamide and methazolamide (diuretic, carbonic anhydrase inhibitor), sulfamethizole (antimicrobial, dihydropteroate synthase inhibitor), desaglybuzole (hypoglycaemic agent), and the 2,3-dihydro-1,3,4-thiadiazoles litronesib (anticancer, kinesin Eg5 inhibitor) and filanesib (anticancer, kinesin spindle protein inhibitor) (Figure 2).

Herein, we report the recent development in the past five years of 1,3,4-thiadiazole compounds as anticancer agents, discussing their synthesis, SAR, antiproliferative mechanism of action, interaction with the biological target, toxicity, and potential clinical implementation in the treatment of tumor diseases. Several thiadiazole compounds, in both free and fused forms, have been reported within the chosen time frame as promising antitumor agents.

## 2. Disubstituted and Trisubstituted 1,3,4-Thiadiazoles with Anticancer Activity

### 2.1. Disubstituted Derivatives

Among the 1,3,4-thiadiazoles endowed with anticancer activity, the most abundant class is represented by the 2,5-disubstituted derivatives.

**Scheme 1:**
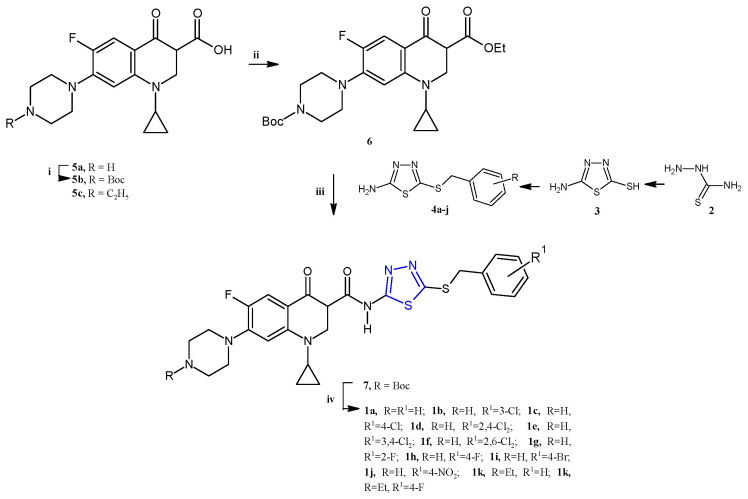
Synthesis of the quinolone-based thiadiazoles **1a–j**. Reagents and conditions: (**i**) Boc_2_O, NaHCO_3_, THF:H_2_O (1:1), r.t., 24 h; (**ii**) ethyl chloroformate, TEA, dry DCM, r.t.; (**iii**) **4a–j**, DCM, r.t., overnight; (**iv**) TFA, DCM 0 °C r.t., 4 h.

Emami et al. recently reported the synthesis and the biological evaluation of a series of ciprofloxacin-derived 1,3,4-thiadiazole analogs: **1a–l** (Scheme 1) [7].

The new quinolone-based thiadiazoles of type **1** were obtained by the reaction of the N-Boc ciprofloxacin **6** (for **1a–j**) or enrofloxacin **5c** (for **1k,l**) with the 5-amino-1,3,4-thiadiazole **4** as reported in Scheme 1 (yields 28–41%). The key intermediates in **4** were synthesized from the heterocyclization of thiosemicarbazide **2** in the presence of carbon disulfide and KOH in absolute ethanol in order to obtain first the 5-amino-1,3,4-thiadiazole-2-thiol **3**, which was subjected to reaction with benzyl halides to afford the desired S-benzyl derivatives **4a–j**.

The ciprofloxacin-based compounds **1a–l** were tested for their in vitro antiproliferative activity against three human cancer cell lines including MCF-7 (breast), A549 (lung), and SKOV-3 (ovarian). The most sensitive cell line, as often observed for thiadiazole derivatives, was found to be the breast cancer cell line MCF-7, against which the new compounds, except for the **1k** derivative, showed IC_50_ values in the range of 3.26–15.7 µM. Indeed, the 4-fluorobenzyl derivatives **1h**,**l** exhibited the highest potency against SKOV-3 and A549 cells, with IC_50_ values of 3.58 and 2.79 µM, respectively.

The flow cytometric analysis of the two most potent compounds, **1e**,**g**, highlighted a cell cycle arrest in sub-G1 phase followed by apoptosis. In particular, compound **1e** showed the highest proapoptotic activity, causing an 18-fold increment in the apoptotic cells at the IC_50_ concentration (3.26 µM). A comet assay was employed in order to evaluate the ability of compounds **1e**,**g**, compared to CF and doxorubicin used as reference drugs, to damage DNA in the cancer cell line MCF-7. The compounds were tested at the three different concentrations, 1, 5, and 10 μM, and the DNA damage was evaluated in terms of tail length, % DNA in tail, and tail moment. The results indicated that compounds **1e**,**g**, even at the lowest tested concentration, displayed a relevant increase in DNA damage. By comparison of the biological results found in the series, some structure–activity relationships can be concluded. The simplest compound **1a** showed good activity against all cell lines, exhibiting a particular selectivity towards MCF-7 cells. The introduction of halogen atoms on the benzyl group, such as Cl or F, significantly improved the anticancer potency against SKOV-3, observing the highest potency for the 4-fluoro-substituted compound (**1h**). Among the halobenzyl analogs (**1b–i**), the 4-bromo-analog **1i** proved to be the most potent against the A549 cell line. These data indicate different effects on the anticancer activity of the substituent on the benzyl group, depending on the cell line studied.

**Scheme 2:**
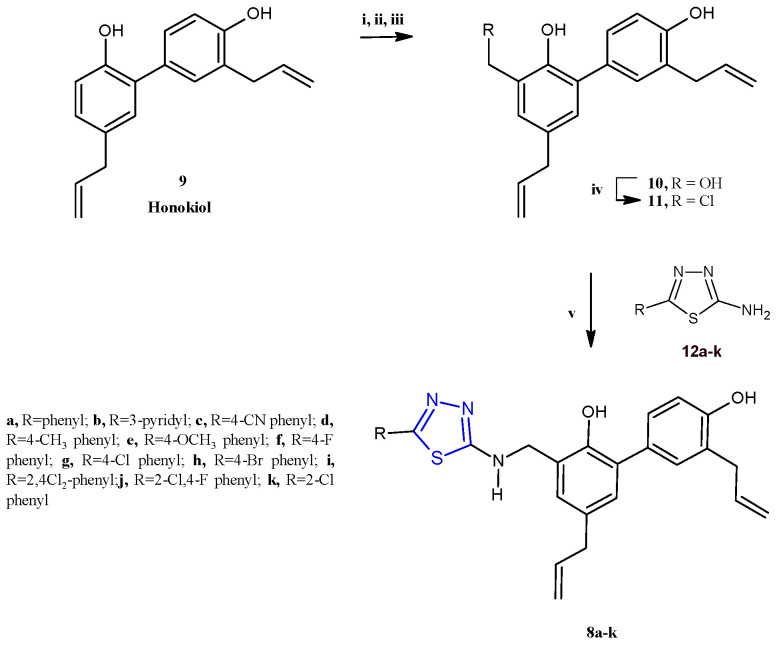
Synthesis of thiadiazole honokiol derivatives **8a–j**. Reagents and conditions: (**i**) I_2_, EtOH/H_2_O (1:9), 50 °C, 12 h; (**ii**) HCHO/NaOH, EtOH/H_2_O (1:1), 50 °C; (**iii**) Zn, AcOH, EtOH, reflux at 78 °C, overnight, (**iv**) SOCl_2_, DCM, 0 °C, 12 h; (**v**) 5-aryl-1,3,4-thiadiazole-2-amine, K_2_CO_3_, acetone, 50 °C, 12 h.

Subsequently, a class of honokiol derivatives **8a–j** (Scheme 2) bearing the 1,3,4-thiadiazole scaffold was described for their interesting anticancer properties against seven cancer cell lines (A549, MDA-MB-231, T47D, MCF-7, HeLa, HCT116, and HepG2) associated with a good toxicity profile [8]. The honokiol **9**, a bioactive compound primarily derived from the species *Magnolia genus*, has been extensively studied for its wide-ranging anticancer properties in both in vitro and in vivo models. Different studies have demonstrated that honokiol modulates various signaling pathways to exert its effects. These include promoting G0/G1 and G2/M cell cycle arrest through the regulation of cyclin-dependent kinases (CDKs), as well as inhibiting epithelial–mesenchymal transition (EMT) by reducing mesenchymal markers while enhancing epithelial markers. Moreover, honokiol effectively suppresses cell migration and invasion by downregulating specific matrix metalloproteinases and activating pathways such as 5′ AMP-activated protein kinase (AMPK) and KISS1/KISS1R signaling. This results in reduced metastasis and enhanced antiangiogenic activity, achieved by decreasing the expression of vascular endothelial growth factor (VEGF) and its receptor (VEGFR) [9]. Compounds **8a–j** were prepared, as reported in Scheme 2, by reacting the honokiol chloromethyl derivative **11** with the suitable 5-aryl-1,3,4-thiadiazole-2-amines **12** at 50 °C, in acetone, in the presence of potassium carbonate as a base (yields 25–76%).

Most of the 1,3,4-thiadiazoles **8a–j** displayed stronger cytotoxicity with respect to the natural precursor honokiol, against all tested cancer cell lines, in particular towards A549 and MDA-MB-231 cells. The most active compounds, **8a**,**d–f**, elicited IC_50_ values ranging from 1.62 to 10.21 μM against all cell panels. In particular, the compound **8a** showed the highest potency against all the seven cancer cell lines with IC_50_ values in the range of 1.62–4.61 μM. The key role of the 1,3,4-thiadiazole scaffold for the pharmacological properties of this series is demonstrated by the drastic drop in activity observed in derivatives in which this heterocycle was replaced by the 1,3,4-oxadiazole isoster, which elicited IC_50_ values ranging from 18.75 to 60.62 μM.

Among the different substituents considered in position 2 of the thiadiazole moiety, the phenyl ring, the para-tolyl, and the para-methoxyphenyl groups exhibited a favorable effect on the anticancer activity, compared with the other substituents. In fact, the derivatives **8a**,**d**,**e** showed IC_50_ values of 1.62, 2.53, and 2.62 μM towards A549 cells, respectively, resulting in significantly more potent effects with respect to the other compounds which elicited IC_50_ values greater than 5.00 μM.

In order to assess the antimetastatic potential of these derivatives, a wound-healing assay on the two cancer cell lines A549 and MDA-MB231 was carried out. Interestingly, the derivative **8a** proved to strongly inhibit migration and invasion in both cell lines. A reduction in migration in A549 and MDA-MB231 cells to 34.77% and 61.57%, respectively, was observed after 48 h of treatment with **8a** at the concentration of 1.25 μM.

Studies on the mechanism of action indicated the induction of cytotoxic autophagy is dose-dependent. The authors hypothesized a mechanism involving the inhibition of the PI3K/Akt/mTor pathway [10] through prediction carried out by the databases of Pharm Mapper, Swiss Target Prediction and Target NeT, and KEGG enrichment analysis.

With the aim of validating this hypothesis on the mechanism of action, the expression levels of PI3K, Akt, mTOR, and their phosphorylated forms were evaluated by Western blot assay in A549 and MDA-MB-231 cells. The results highlighted an important dose-dependent decrease in the expression of p-PI3K, p-Akt, and p-mTOR proteins, in A549 and MDA-MB-231 cells, after **8a** treatment.

The phosphatidylinositol 3-kinase (PI3K)/AKT/mTOR signaling pathway is frequently activated in most human cancers. It is widely recognized for its critical involvement in various cellular processes such as cell proliferation, adhesion, migration, invasion, metabolic regulation, survival, and angiogenesis [11]. Given that numerous transformative events in cancer are driven by heightened signaling in the PI3K/Akt pathway, Akt is considered a promising target for developing new therapies against various tumor types.

**Figure 3:**
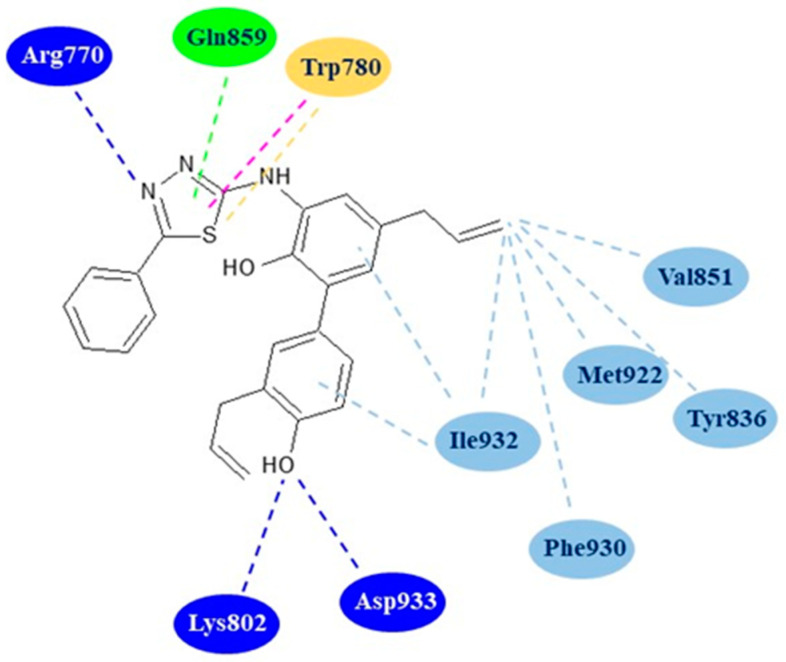
Two-dimensional binding mode of thiadiazole **8a** in the active site of PI3Kα. Blue dotted lines: hydrogen bonds; light blue dotted lines: hydrophobic interactions; green dotted line: carbon hydrogen bond; purple dotted line: Pi–Pi T-shaped interaction, yellow dotted line: Pi–sulfur interaction.

Docking studies on the most active derivative **8a** were performed to evaluate its binding mode with the active site of PI3Kα (PDB code: 4ZOP). Interestingly, the results highlighted that the compound **8a** assumed a conformation very similar to that reported for the known PI3Kα inhibitor [(2S,3R)-N1-(8-(tert-butyl)-4,5-dihydro thia-zolo [4,5-h]quinazolin-2-yl)-3-methylpyrrolidine-1,2-dicarboxamide]. The derivative **8a** showed, in silico, a good affinity towards the kinase forming three hydrogen bonds with the residues Arg770, Lys802 and Asp933 of the active site. Additionally, the complex of the compound **8a** with the protein is further stabilized by numerous hydrophobic interactions with the residues Trp780, Tyr836, Val851, Gln859, Met922, and Ile932 (Figure 3).

The major flaw of this study concerns the lack of confirmation of the mechanism of action with specific assays on the enzyme.

**Scheme 3:**
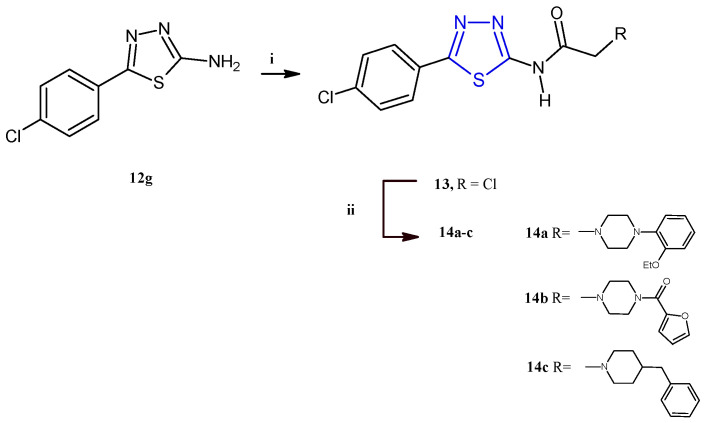
Synthesis of derivatives **14a–c**. Reagents and conditions: (**i**) CH_3_COONa, acetone, chloroacetyl chloride, 0 °C, 1 h; (**ii**) dry benzene, suitable piperazine, TEA, reflux, 16–20 h.

El-Masry and coworkers described the synthesis and the evaluation of the anticancer activity of 5-(4-chlorophenyl)-1,3,4-thiadiazoles combined with other heterocycles including pyridinium, substituted piperazines, benzyl piperidine, and aryl aminothiazoles [12]. Among the fifteen compounds reported, only the derivatives **14a–c** (Scheme 3) were cytotoxic against MCF-7 and HepG2 cancer cells with an IC_50_ range between 2.32 and 8.35 μM. From the analysis of the results, it is evident that the introduction of a piperazine ring through an acetamide linker is advantageous for the antiproliferative activity of this class of compounds. Additionally, the antitumor potency was influenced by the substituents on N4 of piperazine, leading the best results for compounds bearing in this position an *o*-ethoxyphenyl (**14a**) or a furoyl (**14b**), with respect to derivatives substituted with alkyl chains like methyl and ethyl or aromatic moieties such as phenyl, tolyl, *p*-methoxyphenyl, or *p*-fluorophenyl. The replacement of the piperazine nucleus with the 4-benzylpiperidine (**14c**) allowed us to achieve the highest potency against the breast cancer cell line MCF-7 (IC_50_ = 2.32 μM). The compounds **14a–c** showed a good selectivity towards cancer cells since they displayed no cytotoxic effect on the normal cell line Vero (IC_50_ values 84–154 μM). Cell cycle analysis, carried out on HepG2 and MCF-7 cells for the most active compounds, **14a** and **14c**, indicated cellular accumulation in the S and G2/M phases, respectively. Flow cytometric investigations, with propidium iodide (PI) and annexin-V-FITC, on the same two cell lines, indicated apoptosis as the main mode of cell death caused by the derivatives **14a**,**c**.

Since the apoptotic pathway in cancer cells is often inhibited through the upregulation of antiapoptotic proteins, such as Bcl-2, and the underexpression of proapoptotic proteins, such as Bax, the effects of these compounds on the Bax/Bcl2 ratio and caspase 9 levels in HepG2 and MCF-7 were investigated. The results elucidated that the apoptotic mechanism involves an increase in the Bax/Bcl2 ratio and in the concentration of the apoptotic protein caspase 9. The most potent anticancer derivative of the series, compound **14c**, was further selected for radiolabeling and biodistribution studies in order to evaluate its ADME parameters in a sarcoma-bearing mice model. Interestingly, the results indicated that the compound **14c** proficiently targets cancer tissue, showing no accumulation in other organs.

The derivatives **14a–c** were synthesized in good yields (73–85%) starting from the intermediate 2-aminothiadiazole **12g**, which, after treatment with chloroacetyl chloride to give the compound **13**, was reacted with the suitable piperazine to give the target derivatives as described in Scheme 3.

**Scheme 4:**
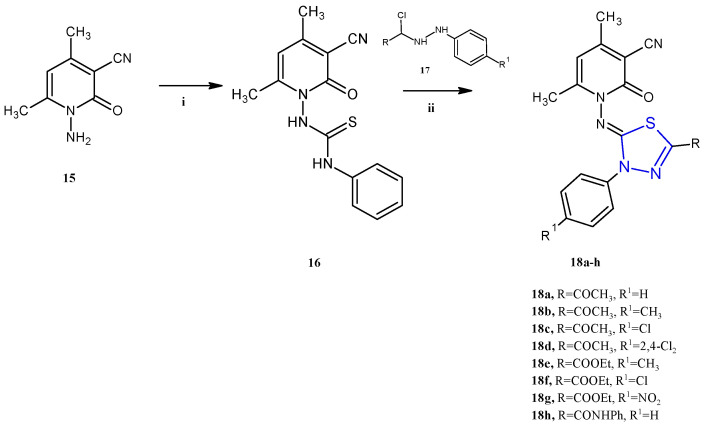
Synthesis of imidazothiadiazoles **18a–h**. Reagents and conditions: (**i**) PhNCS, DMF, r.t.; (**ii**) EtOH, TEA, reflux 6 h.

Many other compounds in which the 1,3,4-thiadiazole scaffold was combined with other heterocycles have been reported in the past five years as anticancer agents. The pyridine derivatives **18a–h** (Scheme 4), described by Gomha and coworkers, showed antiproliferative activity against two cancer cell lines, the human colon carcinoma HCT-116 and the hepatocellular carcinoma Hep-G2 (IC_50_ 2.03–37.56 μM) [13]. In this class of compounds, the antitumor activity appears to be influenced by the substituents on the phenyl or thiadiazole ring. In particular, the highest activity was found with electron-withdrawing substituents like, in descending order of activity, the amido group (**18h**), nitro group (**18g**), or chloro atom (**18f**). Conversely, the introduction of an electron-donating group in the para position of the phenyl ring, such as a methyl (**18b** and **18e**), was detrimental for the cytotoxicity. With regard to the mechanism of action, the authors report only a docking study, that would highlight a good affinity towards the protein Epidermal Growth Factor Receptor Tyrosine Kinase Domain (EGFR TK). In particular, the compound **18h** was found to exhibit the best binding affinity with a ∆G value of −7.1. Unfortunately, no specific study on the enzyme has been carried out to validate this hypothesis.

The derivatives **18a–h** were successfully synthesized through a reaction between the 1-(3-cyano-4,6-dimethyl-2-oxopyridin-1(2H)-yl)-3-phenylthiourea **16** with the hydrazonyl chlorides **17a–h** using ethanol as a solvent and TEA as a catalyst (yields 73–86%). The mechanism of the reaction involved the alkylation of the thiol group of the thiosemicarbazone moiety, followed by an intramolecular cyclization and the elimination of an aniline molecule.

In turn, the key intermediate **16** was obtained by the reaction of phenyl isothiocyanate with the pyridinone-3-carbonitrile **15** in the presence of a catalytic amount of KOH in absolute ethanol as a solvent, as described in Scheme 4.

**Scheme 5:**
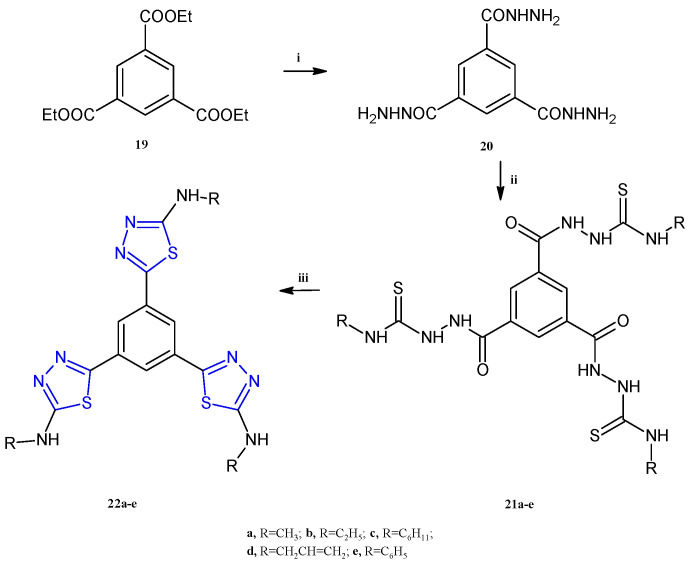
Synthesis of tris-thiadiazoles **22a–e**. Reagents and conditions: (**i**) Ethanol, reflux, 4 h; (**ii**) isothiocyanate derivative, ethanol, reflux, 6 h, (**iii**) sulfuric acid, 0 °C, 30 min, r.t., 16 h.

A class of compounds bearing a central aromatic core trisubstituted with three equal thiadiazole rings, **22a–e** (Scheme 5), were reported as anticancer agents against the HepG-2, MCF-7, and HCT-116 cell lines, able to inhibit the lysine-specific histone demethylase 1A (LSD1, also known as KDM1A) [14]. LSD1 is a protein involved in the removal of marks on histone H3 lysine 4 (H3K4), ubiquitously overexpressed in different cancers including breast, gastric, prostate, hepatocellular, and many others. The important role of this enzyme in differentiation and the increase in proliferation, migration, and invasiveness in cancer cells, has made it a valuable target for the development of antitumor compounds [15]. The highest potency, as often observed for the thiadiazole compounds, was found against the breast cancer cell line MCF-7 with IC_50_ values in the range of 1.52–28.1 μM. The compound **22d**, bearing a propenyl group at the nitrogen atom of the amino group, exhibited the most potent antiproliferative activity against MCF-7 and HCT-116, eliciting IC_50_ values of 1.52 μM and 10.3 μM, respectively. The best candidate for liver cancer was the compound **22a**, which showed an IC_50_ of 6.47 μM. Remarkable LSD1 inhibition was found for all compounds **22a–e** (IC_50_ 0.04–0.45 μM), with the highest potency observed in **22d**. The latter, considered the most promising compound among the synthesized derivatives, has been used for cell cycle analysis and apoptosis detection in MCF-7 cells. The results of the annexin V-FITC and PI apoptosis assay highlighted an arrest at the G2/M phase and DNA fragmentation causing a 114-fold increase in early apoptotic cells. The compound **22d** was evaluated to determine its target selectivity against the structurally similar membrane-bound human enzymes hMAO-A and hMAO-B. The screening was conducted at a concentration of 10 µM and the results from the inhibitory assay demonstrated that the derivative **22d** exhibits molecular selectivity for LSD1 over the MAO isoforms, exceeding the selectivity of standard reference MAO inhibitors.

In order to better understand the mechanism of LSD1 inhibition and to clarify the binding mode of these derivatives in the LSD1 site, a molecular modeling study was carried out on the derivative **22d** using the X-ray crystal structure with PDB code 5YJB.

**Figure 4:**
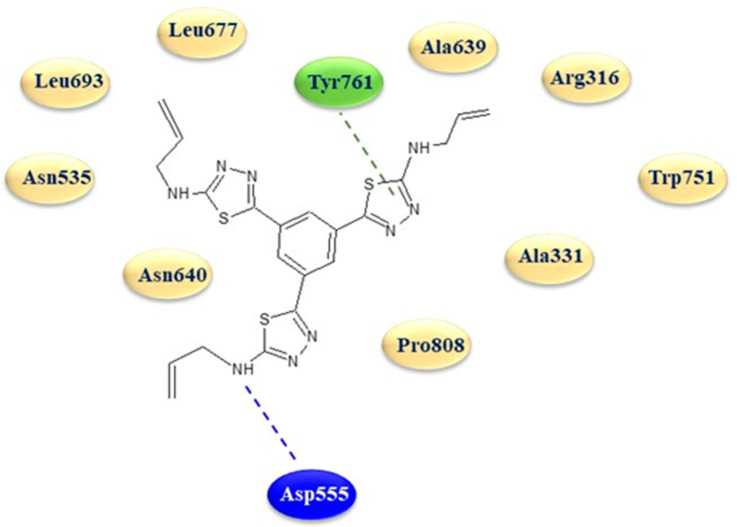
Two-dimensional binding mode of thiadiazole **22d** in the active site of LSD1. Blue dotted line: hydrogen bond; green dotted line: arene interactions.

The in silico analysis revealed a strong affinity of the 1,3,4-thiadiazole **22d** with the protein active site, characterized by two key interactions, typical of LSD1 inhibitors, with the amino acid Asp555 via a hydrogen bond with a terminal amino group on a thiadiazole ring and with Tyr761 through an interaction with one of the three thiadiazole rings (Figure 4).

The synthetic procedure adopted for the synthesis of the compounds **22a–e** is depicted in Scheme 5. In detail, the triheterocyclic derivatives **22a–e** were obtained starting from the triethyl benzene-1,3,5-tricarboxylate **19** which was treated with hydrazine hydrate to afford the benzene-1,3,5-tricarbohydrazide **20**. The following condensation of the derivative **20** with a variety of alkyl-and aryl-isothiocyanates in refluxing ethanol gave the suitable intermediates **21a–e** (87–90% yields). Finally, the desired compounds **22** were obtained in good yields (83–85%) through intramolecular cyclization of the derivatives **21** in sulfuric acid at 0 °C.

The anticancer activity of recently synthesized thiadiazole derivatives was attributed to the inhibition of the human epidermal growth factor receptors (HERs), which are receptor protein tyrosine kinases (PRTKs), that, affecting the secretion of pro-angiogenesis factors, cause an acceleration of cancer development [16]. Among the different HERs, EGFR and HER-2 are highly expressed in various cancer types including breast cancer [17].

Given that many cancers tend to develop resistance to single-target EGFR inhibitors in clinical settings, dual inhibitors designed to simultaneously target EGFR and HER2 have been introduced to enhance therapeutic efficacy, minimize drug resistance and interactions, and promote better patient compliance [18]. 1,3,4-Thiadiazoles of type **29**, differently substituted on the phenyl ring at position 2 of the thiadiazole scaffold, were reported by Jiang et al. as EGFR/HER-2 dual target inhibitors for the treatment of breast and lung cancer [19].

**Scheme 6:**
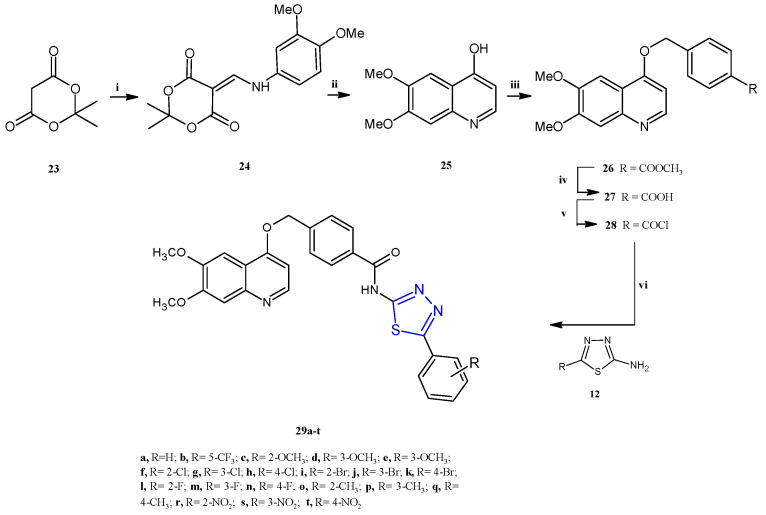
Synthesis of N-(1,3,4-thiadiazol-2-yl)benzamide derivatives **29a–t**. Reagents and conditions: (**i**) trimethyl orthoformate, isopropanol, 85 °C, 0.5–1.5 h; di-methoxyaniline, 85 °C, 3–5 h; (**ii**) phenyl ether, 190 °C, 3–5 h; (**iii**) K_2_CO_3_, methyl 4-(chloromethyl)benzoate, DMF, 60 °C, 6–8 h; (**iv**) NaOH, H_2_O, 45 °C, 2–3 h; (**v**) SOCl_2_, CH_3_Cl, DMF, 55 °C, 5–8 h; (**vi**) TEA, DCM, 0–5 °C, 8–10 h.

The synthetic procedure adopted for the preparation of the N-(1,3,4-thiadiazol-2-yl)benzamide derivatives **29a–t** is depicted in Scheme 6. A two-step condensation between 2,2-dimethyl-1, 3-dioxane-4, 6-dione **23**, trimethyl orthoformate, and 3,4-dimethoxyaniline allowed us to obtain the enamine intermediate **24**. The subsequent cyclo condensation in diphenyl ether gave the compound **25**, which underwent an *O*-alkylation reaction with methyl 4-chloromethyl benzoate in DMF to afford the compound **26**, which was hydrolyzed, in the presence of NaOH, to obtain the corresponding carboxylic acid **27**. The acylchloride compound **28** was easily prepared by the reaction of the acid **27** with SOCl_2_. Finally, the target compounds **29a–t** were achieved in moderate yields (38–53%) through the condensation, in CH_2_Cl_2_ in the presence of trimethylamine, of the compound **28** with the suitable 1,3,4-thiadiazoles of type **12** previously prepared from the corresponding benzoic acid with thiosemicarbazide in POCl_3_.

An in vitro assay performed on MCF-7, SK-BR-3, A549 and H1975 highlighted a strong cytotoxicity in all the tested cancer cell lines for the bromophenyl substituted derivatives **29i–k** (Scheme 6) with IC_50_ values in the range of 0.77–3.43 μM (Table 1). With the aim to verify the antiproliferative effect of the most potent compound, **29i**, an immunofluorescence analysis was performed to evaluate the expression of cytochrome C in cells. An increase in the content of cytochrome C was found in the cytoplasm as the concentration of **30i** increases, with a shift of cytochrome C to the nucleus, which leads to apoptosis, at 0.8 μM. Additionally, trough specific assays, and using reacting oxygen probes to investigate the expression level of ROS, the strong cytotoxic effect on SK-BR-3 viability (IC_50_ = 0.77 μM) of the thiadiazole **29i** was further explored. The compound was found able to cause cell apoptosis by promoting ROS production.

An in vitro kinase assay was performed to evaluate the inhibitory activity of this class of compounds against the four HER family members: EGFR, HER-2, HER-3, and HER-4. The results demonstrated that most of the compounds effectively inhibited EGFR and HER-2, while showing no inhibitory activity towards HER-3 and HER-4. Concerning the EGFR/HER-2 dual-target inhibition, the activity is influenced by the substituent R on the phenyl ring. In particular, substituents as hydrogen (**29a**), trifluoro-methyl (**29b**), a methoxy group (**29c–e**), or a nitro group (**29r–t**) are not advantageous for kinase inhibition. On the contrary, derivatives bearing a halogenated phenyl showed higher potency, exhibiting increasing activity with increasing halogen size, according to the following order: brominated compounds **29i–k** > chlorinated compounds **29f–h** > fluorinated compounds **29l–n**.

Within the first subclass, the *o*-bromophenyl derivative **29i** showed the best enzyme inhibition to EGFR and HER-2, with IC_50_ values of 29.30 nM and 55.69 nM, respectively.

To investigate the effects of the derivative **29i** on breast cancer angiogenesis, an ELISA assay was performed in order to evaluate the content of the pro-angiogenesis factors VEGF and bFGF in the supernatant of SK-BR-3, and a significant reduction in both factors was found after the treatment with **29i**. This result was verified in vitro in human umbilical vein endothelial cells (HUVEC) by performing tube formation experiments to detect the effect of **29i** on angiogenesis. The study highlighted a low toxicity to HUVEC and a strong inhibition of tubule formation. Importantly, the inhibitory effect on angiogenesis, as well as the antiproliferative activity and the low toxicity of the compound **29i**, were confirmed in vivo in a chicken embryo allantoic membrane (CAM) assay and an SK-BR-3 cell xenograft model, respectively.

Furthermore, potential interactions between the derivative **29i** and the EGFR (PDB ID: 1M17) and HER-2 (PDB ID: 3PP0) proteins were investigated through docking analysis. Regarding EGFR, the docking configuration of **29i** revealed several key interactions: both aromatic rings of the quinoline scaffold formed arene-H conjugates with the residue Phe771, while the thiadiazole moiety similarly engaged in an arene-H conjugate with Val702. Additionally, the sulfur atom in the thiadiazole group established a strong hydrogen bond with Thr766, and the bromine atom on the lateral benzene ring formed a halogen bond with Asp831. Similarly, for HER-2, arene-H conjugates were observed between the quinoline aromatic rings and Val734, underscoring the compatibility of the quinoline structure with the binding site. Other weaker interactions were also identified, such as those involving the lateral benzene ring and Leu712, as well as the bromine atom and Ile767. These interactions collectively contribute to the favorable binding of **29i** within the HER-2 active site. A Western blot confirmed the in silico study results, identifying the ability of **29i** in inhibiting the phosphorylation of EGFR and HER-2.

Recently, Serag and co-workers designed and synthesized a new series of 1,3,4-thiadiazole hybrids of type **32** as EGFR inhibitors [20]. Among the new derivatives, the compounds **32a**,**d** exhibited the highest antiproliferative activity against the HePG-2 and MCF-7 cancer cell lines with IC_50_ values ranging from 3.31 to 9.31 µM. The in vitro evaluation of the EGFR inhibitory effect of the compounds **32a,d** indicated a strong enzymatic inhibition with IC_50_ values of 0.08 and 0.30 µM, respectively. Cell cycle analysis performed for the thiadiazole **32a** in MCF-7 cells identified an arrest at G2/M phase and a proapoptotic mechanism involving an increase in the Bax/Bcl-2 ratio, and in turn increases in the level of caspases 6, 7, and 9.

**Scheme 7:**
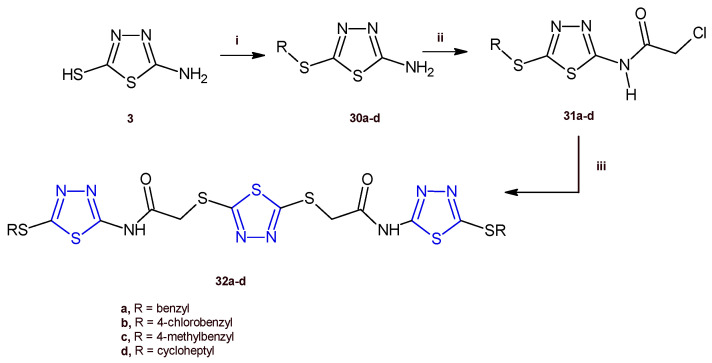
Synthesis of the 1,3,4-thiadiazoles **32a–d**. Reagents and conditions: (**i**) appropriate alkyl halide, K_2_CO_3_/acetone, r.t.; (**ii**) chloroacetyl chloride, K_2_CO_3_/DMF, r.t.; (**iii**) 1,3,4-thiadiazole-2,-dithiol, K_2_CO_3_/acetone, r.t.

The compounds **32a–d** were prepared by adopting the synthetic route depicted in Scheme 7. The thiadiazoles **30a–d** were obtained by stirring, at room temperature, the suitable alkyl halide, with the compound **3**, prepared as previously reported (Scheme 1). The reaction between the derivatives **30a–d** with chloroacetyl chloride allowed us to produce the 2-chloroacetamides **31a–d**, which underwent a nucleophilic substitution reaction with 1,3,4-thiadiazole-2,-dithiol to give the target compounds **32a–d** in good yields (63–70%).

In order to understand the binding mode of the compound **32a** into the EGFR ATP-binding site, a molecular-docking investigation was carried out employing the protein structure with PDB code 4WKQ. The compound was able to establish two hydrogen bonds with the residues Met793 and Lys728 involving the oxygen of the acetamide moiety and the thiol group.

**Figure 5:**
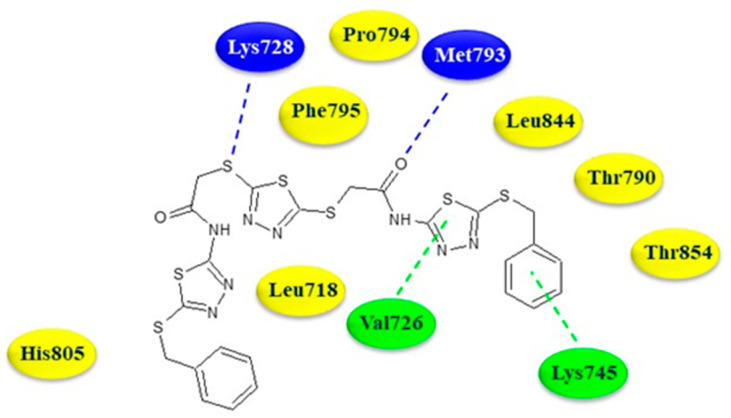
Two-dimensional binding mode of thiadiazole **32a** in the ATP-binding site of EGFR. Blue dotted lines: hydrogen bonds; green dotted line: arene interactions. In yellow the aminoacidic residues involved in hydrophobic interactions.

The thiadiazole ring and phenyl ring of benzylthio moiety formed arene–hydrogen and arene–cation interaction with Val726 and Lys745, respectively. Additionally, numerous hydrophobic interactions with Leu718, Thr790, Pro794, Phe795, His805, Thr854, and Leu844 contribute to strengthening the affinity towards the active site (Figure 5).

**Scheme 8:**
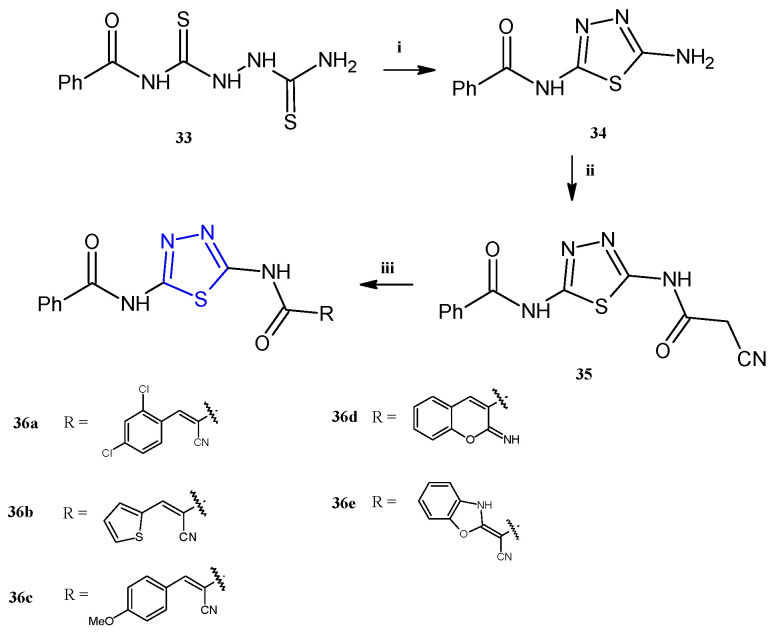
Synthesis of thiadiazoles **36a–e**. Reagents and conditions: (**i**) acetic acid, reflux, 3 h; (**ii**) ethyl cyanoacetate, TEA, absolute ethanol, stirring, 3 h; (**iii**) method A: appropriate aldehyde (for **36a–c**) or 2-(4-methoxy-benzylidene) malonotrile (for **36d**), absolute ethanol, piperidine, reflux, 3 h; method B: KOH, DMF, stirring, 30′, carbon disulfide, methyl iodide, r.t., 3 h, o-aminophenol, dioxane, TEA, reflux, 4 h (for **36e**).

The thiadiazole derivatives **36a–e** (Scheme 8) were recently described for their antiproliferative activity against human breast (MCF-7), colon (HCT-116), prostate (PC-3), and liver (HepG2) cancer cell lines, with particular selectivity towards breast cancer (IC_50_ 5.51–9.48 μM) [21]. Unfortunately, some of the compounds, the derivatives **36a**,**b,d**, were toxic to the normal fibroblasts WI-38 with IC_50_ values in the range of 9.18–29.35 μM. For the derivatives **36c**,**e**, which exhibited a good selectivity towards cancer cells, further investigations in breast cells (MCF-7) were conducted with the aim of evaluating their effect on the cell cycle distribution and the mechanism of cell death. The reported data highlighted different effects of the two compounds: in particular, the derivative **36e** stopped the cell cycle at G2/M and induced early apoptosis, while **36c** blocked the sub-G1 phase inducing necrosis.

The compounds **36a–e** were successfully prepared from the same starting material: the N-(5-(2-cyanoacetamido)-1,3,4-thiadiazol-2-yl)benzamide **35**, as reported in Scheme 8. This key molecule was obtained in high yields (75%) through a three-step synthesis involving the following: (**i**) the reaction of benzoylisothiocyanate with thiosemicarbazide in dry acetonitrile to obtain the N-(5-amino-1,3,4-thiadiazol-2-yl) benzamide **33**; (**ii**) cyclization in acetic acid to afford the aminothiadiazole **34**; and (**iii**) final reaction of the intermediate **34** with ethyl cyanoacetate in the presence of a catalytic amount of trimethylamine to gain the thiadiazole benzamide **35**. The Knoevenagel condensation of the latter with different aldehydes, in refluxing ethanol in the presence of a catalytic amount of piperidine, allowed us to obtain the target compounds **36a–e** in excellent yields (65–84%).

Molecular docking analysis carried out on the compounds **36a–e** suggested a possible mechanism of CDK1 inhibition, which, however, has not been confirmed with a specific enzymatic assay.

**Figure 6:**
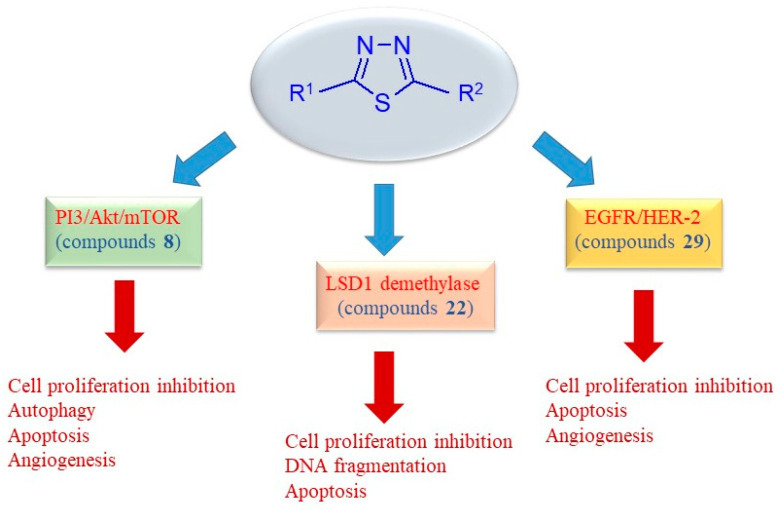
Schematic representation of key anticancer mechanisms of uncondensed thiadiazoles.

The main signaling pathways involved in the antitumor mechanism of action of the 2,5-disubstituted thiadiazole derivatives are summarized in Figure 6.

Other classes of uncondensed 2,5-disubstituted 1,3,4-thiadiazoles have been reported as anticancer agents in the past five years, but the antiproliferative activity described is very low; therefore, they are not discussed in this review, which focuses on promising antitumor compounds with a thiadiazole structure [22,23,24,25].

**Figure 7:**
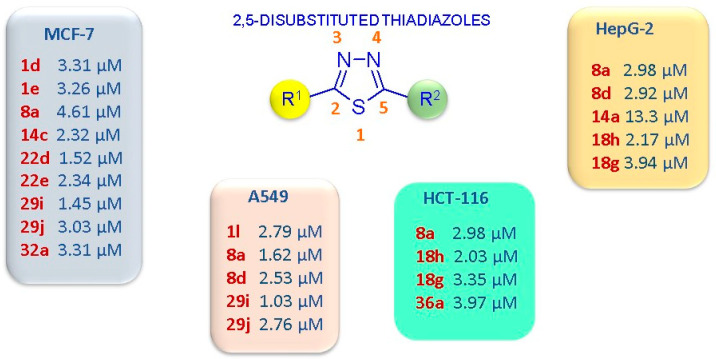
Schematic representation of the 2,5-disubstituted thiadiazoles with the highest anticancer potency, expressed as IC_50_ values, and the most sensitive cancer cell lines.

In Figure 7, the most potent anticancer compounds with a 2,5-disubstituted thiadiazole scaffold are summarized.

### 2.2. Trisubstituted Derivatives

**Scheme 9:**
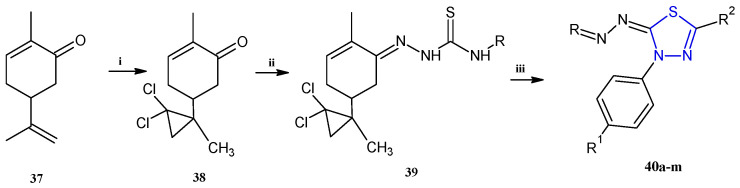
Synthesis of thiadiazoles **40a–m**. Reagents and conditions: (**i**) NaOH, PTC, CHCl_3_; (**ii**) H_2_SO_4_, EtOH, reflux, 3 h, thiosemicarbazide derivatives; (**iii**) EtOH, Et_3_N, reflux, 3 h, Method A: diarylnitrilimines (DANI); Method B: N-aryl-C-ethoxycarbonylnitrilimines (NACE).

Trisubstituted thiadiazole derivatives described as anticancer agents in the last 5 years were not as numerous and promising as the disubstituted analogues. The only class of compounds described, the derivatives **40a–m** (Table 2), were obtained through a three-step synthesis, which begins with a dichloro-cyclopropanation reaction, to obtain the derivative **38**, followed by condensation with thiosemicarbazide to give the intermediate **39**, to end with a 1,3-dipolar cycloaddition reaction with different nitrilimines to give the targeted compounds **40a–m** in yields ranging from 47% to 85% (Scheme 9) [26,27].

**Table 2:** Trisubstituted thiadiazole derivatives **40a–m** described as anticancer agents.

Compound	R	R^1^	R^2^
**40a**	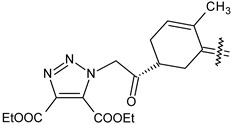	H	4-CH_3_ phenyl
**40b**	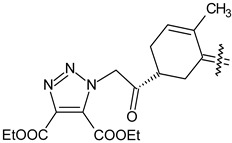	H	4-Cl phenyl
**40c**	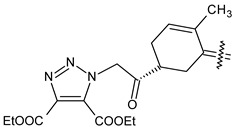	H	4-NO_2_ phenyl
**40d**	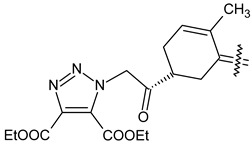	CH_3_	COOC_2_H_5_
**40e**	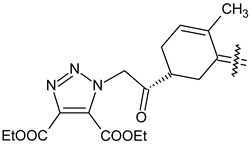	Cl	COOC_2_H_5_
**40f**	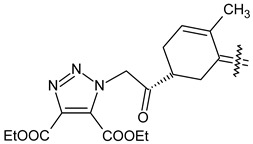	NO_2_	COOC_2_H_5_
**40g**	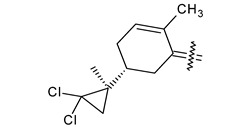	H	phenyl
**40h**	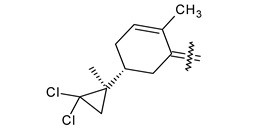	H	4-CH_3_ phenyl
**40i**	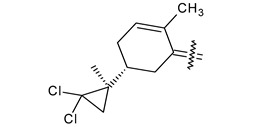	H	4-Cl phenyl
**40j**	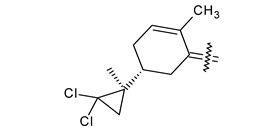	H	4-NO_2_ phenyl
**40k**	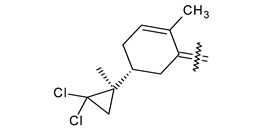	CH_3_	COOC_2_H_5_
**40l**	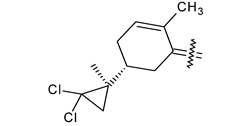	Cl	COOC_2_H_5_
**40m**	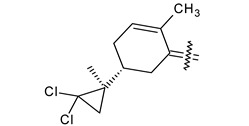	NO_2_	COOC_2_H_5_

The thiadiazoles **40a–m** were evaluated only for their in vitro antiproliferative activity against the human cancer cell lines HT-1080, A-549, MCF-7, and MDA-MB 231 without any further assays on the cell cycle modulation or apoptotic effects. Moderate-to-weak anticancer activity was found in the tumor cells tested, with IC_50_ values ranging from 16.12 to 61.81 µM.

The authors reported a docking analysis of the compounds **40** on the antiapoptotic proteins Bcl-2, Bcl-XL, and caspase-3, which indicated a good affinity of the compounds **40a** and **40i** towards Bcl-2 and caspase-3, respectively. However, no experimental data on apoptosis were reported by the authors in order to corroborate the theoretical analyses reported.

## 3. Imidazo [2,1-*b*][1,3,4]Thiadiazoles with Anticancer Activity

Among the fused 1,3,4-thiadiazole derivatives endowed with anticancer activity, the most representative class is constituted by the imidazo [2,1-*b*][1,3,4]thiadiazoles [28].

**Scheme 10:**
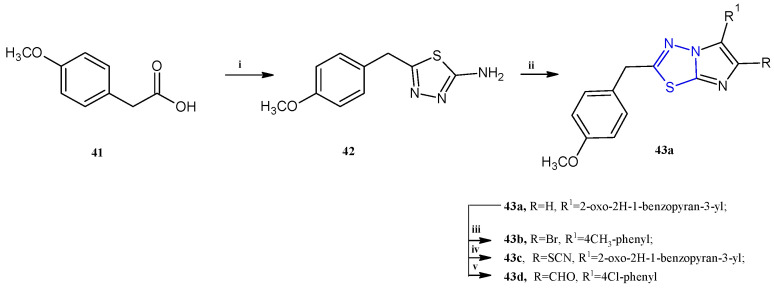
Synthesis of imidazo [2,1-*b*][1,3,4]thiadiazoles **43a–d**. Reagents and conditions: (**i**) thiosemicarbazide, H_2_SO_4_, 60–70 °C, 8 h; (**ii**) EtOH, bromoacetyl derivative, 12 h, K_2_CO_3_; (**iii**) AcOH, Br_2_, AcONa, 1 h; (**iv**) AcOH, Br_2_, KSCN, 4 h; (**v**) DMF, POCl_3_, 80 °C, 4 h.

Choodamani et al. reported a class of imidazothiadiazoles of type **43** (Scheme 10) as cytotoxic agents against the three cancer cell lines L1210, CEM, and HeLa cells [29].

The synthesis of this class of compounds was carried out as reported in Scheme 10. The key intermediate **42** was obtained by reacting 4-methoxyphenylacetic acid **41** with thiosemicarbazide in sulfuric acid. The 6-aryl-substituted **43a** was achieved by refluxing in ethanol the derivative **42** with the suitable 2-bromoketone. The other compounds of the series, **43b–d**, were obtained by electrophilic substitutions on imidazo [2,1-*b*][1,3,4]thiadiazoles **43a** (yields: 58–70%).

Here, we report the most active compounds of the series, derivatives **43a–d**, which showed IC_50_ values in the range of 0.78–90.0 μM (Table 3). In this series, the trisubstituted derivatives proved to be more potent with respect to the disubstituted compounds. Among the compounds 2,6-disubstituted, only the derivative **43a** showed good cytotoxicity, selectively against human T-lymphocyte CEM cells, with an IC_50_ of 5.0 μM, exhibiting a weak activity against HeLa and L1210 cells (IC_50_ of 70.0 and 90.0 μM, respectively). The presence of a bromine atom at the fifth position of the imidazothiadiazole scaffold is not particularly advantageous for the antitumor property; in fact, the most active brominated derivative, **43b**, showed only moderate cytotoxicity against the three tested cell lines (IC_50_ 13.0–33.0 μM).

The best results have been found introducing at the C5 position a thiocyanate (**43c**) or a formyl group (**43d**) leading to compounds with IC_50_ values ranging from 0.78 μM to 1.6 μM.

The most potent imidazothiadiazoles, **43c**,**d**, were further assayed in order to extend the antiproliferative studies against other cancer cell lines including Jurkat (acute T-cell leukemia) and CCRF-CEM (acute lymphoblastic leukemia); moreover, the safety profile was investigated in Hs27 cells (non-cancerous fibroblasts). The two compounds significantly inhibited cell proliferation in these cancer cells, with IC_50_ values ranging from 1.65 to 4.73 μM. Unfortunately, the derivative **43d** also exhibited cytotoxicity towards normal cells, with an IC_50_ of 2.36 μM. In contrast, the derivative **43c** demonstrated better selectivity, showing an IC_50_ of 31.45 μM against the normal Hs27 cells.

**Table 3:** Anticancer activity of imidazothiadiazoles **43a–d** against CEM, HeLa, and L1210 cancer cell lines.

Comp		IC_50 (µM)_	
	CEM	HeLa	L1210
**43a**	5.0	70	90
**43b**	13	18	33
**43c**	0.79	0.78	1.6
**43d**	0.94	1.3	1.1

Further studies conducted in the Jurkat cell line on the most potent compound **43** indicated its ability to cause cell death through phosphatidylserine externalization, caspase-3 activation, and consequent cellular apoptosis. Additionally, cell cycle analysis highlighted the increase in cell population in G2-M, suggesting interference with cytokinesis.

**Figure 8:**
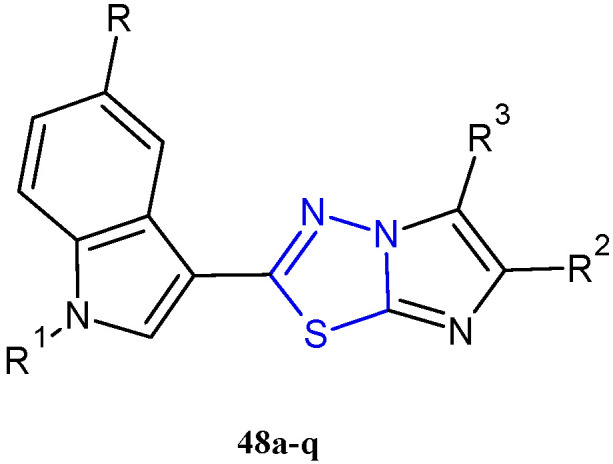
Chemical structures of 3-(imidazo [2,1-*b*][1,3,4]thiadiazol-2-yl)-1*H* indole analogues **48a–q** with potent antiproliferative activity.

In recent years, using a hybridization approach, which consists of the combination of two or more pharmacophores in order to obtain new derivatives characterized by a synergism of the biological activities as well as a greater ability to overcome drug-resistance mechanisms and lower side effects, numerous 3-(imidazo [2,1-*b*] [1,3,4]thiadiazol-2-yl)-1H indole analogues have been synthesized and tested as anticancer agents. The combination of the two relevant bioactive moieties, thiadiazole and indole, has provided us with derivatives of type **48** (Figure 8, Table 4) with potent anticancer properties [30,31,32,33].

**Scheme 11:**
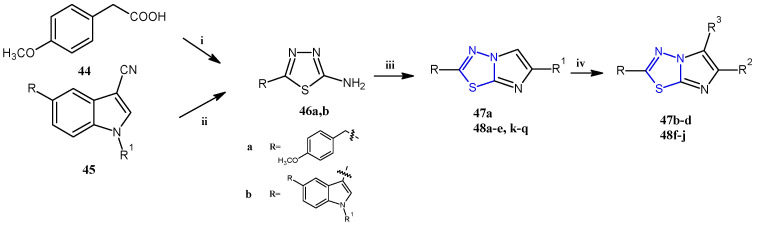
General synthesis of imidazothiadiazoles **47** and **48**. Reagents and conditions: (**i**) H_2_SO_4_ conc, 60–70 °C, 8 h; (**ii**) TFA, 60 °C, 3.5 h; (**iii**) β-bromoacetyl compounds, anhydrous ethanol, reflux 24–48 h, (**iv**) POCl_3_, DMF, 0–5 °C, DMF, 70 °C, 5 h.

The imidazothiadiazole derivatives **47** and **48** were prepared, as reported in Scheme 11, from the key intermediate **46**, which was easily obtained in excellent yields (98–100%) by treating, with thiosemicarbazide, the corresponding carboxylic acid **44** or indole-3-carbonitrile **45**, at 60 °C in sulfuric acid or trifluoroacetic acid, respectively. The reaction of the aminothiadiazole **46** with the appropriate β-bromoacetyl compounds in refluxing ethanol gave the bicyclic derivatives **47a** and **48a–e,k–q** (55–80%). The compounds substituted in position 5 with a bromine atom (**47b**) or a thiocyanate group (**47c**) were obtained by reacting **47a** with bromine or potassium thiocyanate in acetic acid. The 5-carbaldehyde derivatives (**47d** and **48f–j**) were instead prepared through a formylation under standard Vilsmeier conditions carried out on the corresponding imidazothiadiazoles that were not substituted (with R^3^ = H).

The imidazo [2,1-*b*][1,3,4]thiadiazoles **48** were initially tested by the National Cancer Institute (NCI; Bethesda, MD) for the evaluation of their antitumor activity. A preliminary screening, according to the NCI protocol, at one dose of 10 µM on a panel of 60 human cancer cell lines belonging to 9 cancer cell types including leukemia, non-small cell lung, colon, central nervous system, melanoma, ovarian, renal, prostate, and breast cancers was carried out on all compounds in **48**. The derivatives **48a,c,j–m**, having satisfied the requirements set by the NCI, were subjected to further tests at 5 different concentrations in order to evaluate their IC_50_ values. The most potent derivatives **48a,c** showed significant in vitro anticancer activity against all tested cell lines, with GI_50_ values ranging from 0.23 to 11.4 µM, and 0.29 to 12.2 µM, respectively [30]. Although less potent than the derivatives **48a,c**, the compounds **48j–m** showed good antiproliferative activity (GI_50_ 0.19–83.6 µM), eliciting a marked selectivity towards MCF-7 with GI_50_ values in the range of 0.29–0.59 µM.

In order to extend the antiproliferative studies to different cancer types not included in the NCI panel, the cytotoxicity of this class of compounds was also evaluated in vitro on a panel of pancreatic ductal adenocarcinoma (PDAC) cells, including SUIT-2, Capan-1, and Panc-1 [31], and on the two peritoneal mesothelioma cell lines MesoII and STO cells [33]. PDAC represents more than 90% of pancreatic cancer cases and, currently is considered one of the deadliest tumors, with a 5-year overall survival rate of 13% [34]. Despite many efforts having been made in the last 10 years to develop new efficacious therapeutic strategies in the treatment of PDAC disease [35,36], currently, surgical resection remains the unique valid curative option for this malignancy.

Diffuse malignant peritoneal mesothelioma (DMPM) presents significant challenges in diagnosis, both clinically and histologically, and is associated with a poor prognosis. The majority of patients achieve better outcomes through a multimodal therapeutic approach, typically involving a combination of surgery and chemotherapy [37].

Interestingly, the compounds **48a–d** exhibited remarkable antiproliferative activity on both cancer types, PDAC and DMPM, with IC_50_ values in the range from 0.85 to 4.86 µM and 0.59 to 5.90 µM, respectively. Notably, the derivatives **48a,b,k** exhibited the highest potency against PDAC cell lines [31] with IC_50_ values in the range of 0.85–1.70 µM, and, for compounds **48a,b**, also against DMPM cells (0.59–2.81 µM) [30].

For a long time, gemcitabine monotherapy has been employed as a first-line treatment for metastatic PDAC and is still a mainstay of PDAC treatment at all stages of this malignancy. Unfortunately, due to the onset of PDAC resistance, the effectiveness of this drug has been significantly reduced [38]. In order to evaluate the ability of the imidazothiadiazoles **48** to circumvent gemcitabine chemoresistance, the cytotoxicity of the compounds **48a–d** was also evaluated in vitro against Panc-1R cells, a gemcitabine-resistant sub-clone obtained by the continuous incubation of Panc-1 with 1 µM of the drug [39]. Notably, all tested compounds proved to be cytotoxic against Panc-1R, with IC_50_ values ranging from 2.2 µM (**48b**) to 3.9 µM (**48d**) [31].

The antiproliferative activity of the most active compounds of the series, **48a,b**, was confirmed using the three-dimensional (3D) model of PDAC-3, Meso II, and STO. Spheroids better representing the real 3D architecture of tumors with respect to the traditional 2D cell cultures provide more reliable results for the evaluation of the effects on tumor growth and cell–cell interactions. Treatment with **48a,b** at a 5× IC_50_ concentration clearly hinder the spheroids’ formation after just 5 days in all models studied.

Given the high metastatic potential of PDAC and DMPM, an effective therapeutic strategy against these malignancies should have an antimigratory effect in addition to antiproliferative activity. Therefore, the ability of compounds **48a,b** to inhibit migration was evaluated by a scratch wound-healing assay in both cancer types. The results highlighted the high ability of the imidazothiadiazole compounds to reduce the rate of cell migration in all the PDAC preclinical models used, as well as in STO cells. In particular, using a concentration equal to 4x IC_50_ of the compounds **48a,b**, after 24 h from the start of the treatment, the percentages of migration in cells treated were reduced from 100% (control) to 33.3% and 32%, respectively, in Panc-1R; 34.9% and 41%, respectively, in SUIT-2; and 25.8% and 20%, respectively, in STO cells.

Significant antimigratory activity was also evident in PDAC cells for the compound **48k**.

**Figure 9:**
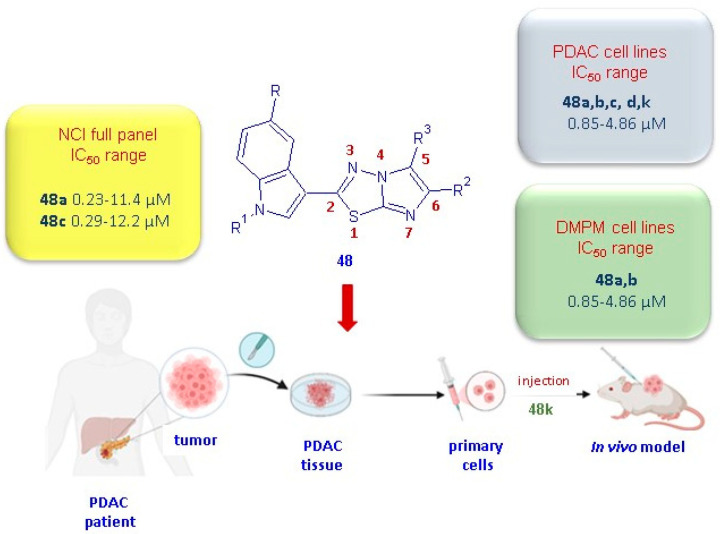
Anticancer properties of the most potent imidazothiadiazoles of type **48**.

Specifically, when compared to the untreated cells considered as 100%, the migration percentage for cells treated with the compound **48k** was 19% in SUIT-2 cells, 27% in the resistant clone PANC-1 GR, and 27% in PDAC-3 primary cells. The antitumor activity observed in in vitro PDAC models for the compound **48k** was confirmed in an in vivo model (Figure 9). Remarkably, the compound **48k** demonstrated a significant effect (*p* < 0.05) on a mice xenograft model after only two weeks of treatment with 25 mg/kg, three times a week.

With the aim of investigating the mechanism of action underlying the promising antiproliferative and antimigratory activity found for the compounds **48**, a high-throughput analysis with the Pamgene tyrosine kinase peptide substrate array (PamChip) and an ELISA assay were carried out. The results identified the non-receptor tyrosine kinase focal adhesion kinase (FAK) as the main target of this class of compounds. Considering the key role of FAK in the control of several cellular processes correlated with many aspects of tumorigenesis, including survival, proliferation, and motility [40], and considering that FAK is often overexpressed in PDAC cells, a mechanism involving FAK inhibition is perfectly congruent with the antiproliferative and antimigratory activity found for the imidazothiadiazoles of type **48**.

## 4. Conclusions

The thiadiazole nucleus represents a valuable scaffold for the development of bioactive molecules endowed with different therapeutic activities [41,42,43,44,45,46].

In this review, we focused on 1,3,4-thiadiazole compounds described for their anticancer activity, which were divided into two classes: unfused compounds and derivatives bearing the thiadiazole ring fused to another heterocyclic ring. Several new thiadiazole derivatives have recently been reported to exhibit antitumor activity attributable to the inhibition of different pathways, and the most representative compounds are listed in Table 5.

Among the thiadiazoles belonging to the first class, the derivatives substituted at positions 2 and 5 of the thiadiazole nucleus are the most abundant and showed the highest antiproliferative potency. Numerous compounds have been found to be very effective especially in the treatment of breast cancer, particularly towards the MCF-7 cell line, exhibiting proapoptotic behavior.

Noteworthily, the derivative **22d** elicited an IC_50_ value of 1.52 µM on the MCF-7 cell line, causing an arrest at the G2/M phase of the cell cycle and DNA fragmentation due to a mechanism involving a strong inhibition of LSD1 (IC_50_ = 0.046 µM). The in-depth studies conducted on this derivative regarding cytotoxicity and mechanism of action provide useful information for the design of new anticancer agents with a thiadiazole structure.

The uncondensed thiadiazole **29i** proved to be very potent against the breast cancer cells MCF-7 and SK-BR-3, showing IC_50_ values of 1.45 µM and 0.77 µM, respectively. In this case, the activity was attributed to the hindrance of EGFR and HER-2 with an IC_50_ of 29.30 nM and 55.69 nM, respectively. Importantly, the value of the compound **29i** as an antiangiogenic and anticancer agent was also confirmed in vivo in a chicken embryo allantoic membrane (CAM) assay and in a mice xenograft model. The investigation of the binding modes of the thiadiazole **29i** inside the active site of the two human epidermal growth factor receptors, EGFR and HER-2, furnished precious details on the key structural features responsible for the interactions between the compound and the proteins. The results highlighted the crucial involvement of the thiadiazole moiety in the interaction with the biological target. These in silico data, together with the SAR considerations of these derivatives, can be exploited for a targeted design of new potent compounds.

For the other disubstituted derivatives, the main shortcoming is, in many cases, the lack of valid computational studies on the targets which can allow researchers to optimize the design of new more potent derivatives. Most of the docking studies reported for this class of compounds, in fact, are often not supported by appropriate studies on the enzyme and therefore are not reliable as a model [13,22,23,24].

Among the condensed 1,3,4-thiadiazole derivatives endowed with anticancer activity, the most representative compounds bear the thiadiazole moiety fused with an imidazole ring. Especially noteworthy are the imidazothiadiazoles **48a,b**, which are substituted in position 2 with an indole scaffold and in position 6 with a thiophene ring. These two compounds reported potent antiproliferative activity against the full NCI panel of cancer cells, as well as against PDAC and DMPM, exhibiting IC_50_ values in the low micromolar to nanomolar range. SAR studies highlighted the importance of the indole nucleus in position 2; in fact, its replacement with other heterocycles as well as its movement to position 6 causes a sudden decrease in antitumor activity. Among the different substitutions considered in position 6, the thiophene ring proved to be the most advantageous.

Interestingly, the compounds **48a**,**b** displayed strong antimigratory and antimetastatic activity in different PDAC cell lines, including SUIT-2, Capan-1, Panc-1, and Panc-1R, and in the DMPM cells STO. Studies on the mechanism of action indicated a marked inhibition of the PTK2/FAK pathway, whose key role in tumorigenesis and in PDAC cancer progression and resistance is widely described.

1,3,4-Thiadiazole derivatives have demonstrated promising anticancer activity in preclinical studies, in particular for the treatment of breast and pancreatic diseases, but, in many cases, their full therapeutic potential remains unexplored due to the lack of bioavailability and pharmacokinetics studies. To unlock their potential as anticancer drugs and in order to optimize the clinical application of thiadiazole compounds, additional in vivo studies would be very helpful to better evaluate both their therapeutic and safety profiles.

On the basis of the significant results described for this heterocycle, the design of new antitumor compounds bearing this pharmacophore is strongly encouraged.

## Figures and Tables

**Table 1:** Cytotoxicity of derivatives **29a–t** on A549, MCF-7, H1975, and SK-BR-3.

Compounds	IC_50_ (µM)			
	A549	MCF-7	H1975	SK-BR-3
**29a**	18.32	19.88	15.68	9.14
**29b**	9.20	17.04	14.09	9.24
**29c**	8.75	11.15	10.12	8.26
**29d**	7.33	16.31	14.81	5.96
**29e**	7.41	18.64	12.02	6.08
**29f**	3.45	7.54	3.91	2.31
**29g**	3.54	7.29	4.39	3.83
**29h**	4.08	8.48	4.13	3.86
**29i**	1.03	1.45	1.08	0.77
**29j**	2.76	3.03	2.83	1.41
**29k**	3.43	3.42	3.14	2.05
**29l**	4.35	7.04	6.12	3.65
**29m**	4.79	9.93	3.52	3.95
**29n**	6.91	8.54	6.99	5.10
**29o**	3.63	7.00	5.08	4.39
**29p**	6.47	8.45	4.14	4.78
**29q**	4.82	7.31	5.51	4.19
**29r**	5.93	7.59	5.81	6.10
**29s**	6.54	11.60	6.41	6.59
**29t**	6.35	10.05	6.13	6.26
**Cabozantinib**	4.66	5.50	3.36	3.88

**Table 4:** Imidazothiadiazole derivatives **48a–q** described as anticancer agents.

Compound	R	R^1^	R^2^	R^3^
**48a**	H	H	tiophen-3-yl	H
**48b**	H	CH_3_	tiophen-3-yl	H
**48c**	F	CH_3_	tiophen-3-yl	H
**48d**	F	H	tiophen-3-yl	H
**48e**	Br	H	tiophen-3-yl	H
**48f**	H	H	C_6_H_5_	CHO
**48g**	H	H	4F-phenyl	CHO
**48h**	H	H	4NO_2_-phenyl	CHO
**48i**	Br	H	2,5-OCH_3_-phenyl	CHO
**48j**	H	H	furan-2-yl	CHO
**48k**	H	CH_3_	furan-2-yl	H
**48l**	Br	CH_3_	furan-2-yl	H
**48m**	F	CH_3_	furan-2-yl	H
**48n**	F	H	furan-2-yl	H
**48o**	Cl	CH_3_	2,3-dihydro-benzo [1,4] dioxin-6-yl	H
**48p**	OCH_3_	H	4NO_2_-phenyl	H
**48q**	F	H	C_6_H_5_	H

**Table 5:** Anticancer activity and biological target of representative thiadiazole compounds.

Compounds	Thiadiazole Class	Anticancer Activity IC_50_ (µM)	Biological Target	Ref.
**1l**	2,5-disubstituted	2.79 (A545)	n.i.	[7]
**8a**	2,5-disubstituted	1.62 (A549)	PI3K/Akt/mTor	[8]
**14c**	2,5-disubstituted	2.32 (MCF-7)	n.i.	[12]
**18h**	2,5-disubstituted	2.03 (HCT-116) 2.17 (HepG-2)	EGFR-TK	[13]
**22d**	2,5-disubstituted	1.52 (MCF-7)	LSD-1	[14]
**29i**	2,5-disubstituted	1.03 (A549) 1.45 (MCF-7) 0.77 (SK-BR-3)	EGFR/HER-2	[19]
**32a**	2,5-disubstituted	3.31 (MCF-7)	EGFR	[20]
**43a**	imidazothiadiazole	3.97 (HCT-116)	CDK-1	[29]
**47c**	imidazothiadiazole	0.79 (CEM) 0.78 (HeLa)	n.i.	[31]
**48a**	imidazothiadiazole	0.23 (MBA-MB 435)	PTK2/FAK	[33]

## Data Availability

No new data were created. Data sharing is not applicable to this article.

## Abbreviations

The following abbreviations are used in this manuscript:

AMPK, 5′ AMP-activated protein kinase; CAM, chicken embryo allantoic membrane; CDKs, cyclin-dependent kinases; CF, ciprofloxacin; DCM, dichloromethane; DMPM, diffuse malignant peritoneal mesothelioma; EGFR TK, Epidermal Growth Factor Receptor Tyrosine Kinase Domain; EMT, epithelial–mesenchymal transition; FAK, focal adhesion kinase; HERs, human epidermal growth factor receptors; HUVEC, human umbilical vein endothelial cells; LSD1, lysine-specific histone demethylase 1A; NCI, National Cancer Institute; PDAC, pancreatic ductal adenocarcinoma; PI, propidium iodide; PRTKs, receptor protein tyrosine kinases; TEA, triethylamine; TFA, trifluoroacetic acid; VEGF, vascular endothelial growth factor; VEGFR, vascular endothelial growth factor receptor.
